# Geological and geochemical characterizations of sediments in six borehole cores from the arsenic-contaminated aquifer of the Mekong Delta, Vietnam

**DOI:** 10.1016/j.dib.2019.104230

**Published:** 2019-07-11

**Authors:** Dang Thuong Huyen, Carlito Baltazar Tabelin, Huynh Minh Thuan, Dang Hai Dang, Phan Thi Truong, Banthasith Vongphuthone, Masato Kobayashi, Toshifumi Igarashi

**Affiliations:** a Earth Resources and Environment, Faculty of Geology and Petroleum Engineering, Ho Chi Minh City University of Technology, 168 Ly Thuong Kiet, Dist. 10, HCMC, Viet Nam; b School of Minerals and Energy Resources Engineering, The University of New South Wales, Sydney, NSW 2052, Australia; c Division of Sustainable Resources Engineering, Graduate School of Engineering, Hokkaido University, Japan; d Division of Sustainable Resources Engineering, Faculty of Engineering, Hokkaido University, Japan

## Abstract

The Mekong Delta, situated between Cambodia and Vietnam, is one of the most productive aquifer systems in the region. In recent years, however, several studies have shown that groundwater in several areas of the delta is highly contaminated with arsenic (As). Although more than 80% of the total area of the Mekong Delta is situated in Vietnam, most of the studies have been conducted on the Cambodian-side of the delta. In this study, borehole core samples were collected around the Tien and Hau Rivers, the two main branches of the Mekong River as it enters Vietnam. We present a raw data collection of the chemical and mineralogical composition of distinct lithological features from six borehole core samples drilled up to a depth of 40 m. The data also include the pH, Eh, EC, As, Si, Al, DOC, dissolved heavy metals (Fe and Mn) and major coexisting ions of leachates obtained by leaching the 34 selected sediment samples in deionized water. The information provided in this paper would be useful as a baseline for reactive transport or geochemical modeling to understand and predict As migration in naturally contaminated aquifers under various conditions. For more insights, the reader is referred to our paper entitled “The solid-phase partitioning of arsenic in unconsolidated sediments of the Mekong Delta, Vietnam and its modes of release under various conditions” Huyen et al., 2019.

**Table:** Specifications table

Subject area	*Geology, Geochemistry*
More specific subject area	*Environmental geochemistry, sediment lithology and composition, leaching and speciation of arsenic*
Type of data	*Table, image (SEM-EDX), figure (Eh-pH diagram)*
How data was acquired	*Geological survey, SEM-EDX, XRD, XRF, ICP-AES, TOC Analyzer, LOI, ion chromatography*
Data format	*Raw, analyzed*
Experimental factors	*Standard preservation procedures until the analyses*
Experimental features	*Chemical composition, total organic carbon, mineralogical composition of sediments and pH, Eh, dissolved Fe, Mn, Al, Si, DOC, major cations (Ca*^*2+*^*, Na*^*+*^*and K*^*+*^*) and major anions (SO*_*4*_^*2−*^*, Cl*^*−*^*, HCO*_*3*_^*−*^*and NO*_*3*_^*−*^*) of the leachates were measured*
Data source location	*An Giang and Dong Thap Provinces of Vietnam*
Data accessibility	*Data is included in this article*
Related research article	*Huyen, D.T., Tabelin, C.B., Thuan, H.M., Dang, D.H., Truong, P.T., Vongphuthone, B., Kobayashi, M. and Igarashi, T., 2019. The solid-phase partitioning of arsenic in unconsolidated sediments of the Mekong Delta, Vietnam and its modes of release under various conditions. Chemosphere 233, 512–523.**https://doi.org/10.1016/j.chemosphere.2019.05.235*

**Table:** 

**Value of the data** • The dataset could be compared with those obtained from the Cambodian-side of the Mekong Delta to further understand not only the extent of arsenic contamination but also the migration of this toxic element through the aquifer. • The dataset could be used as a benchmark for other researchers engaged in the reactive transport or geochemical modeling of arsenic in aquifer systems. • The dataset could help explain why certain parts of the Mekong Delta are highly contaminated with arsenic and help local agencies to identify high-risk areas.

## 1 Data

**Fig. 1:**
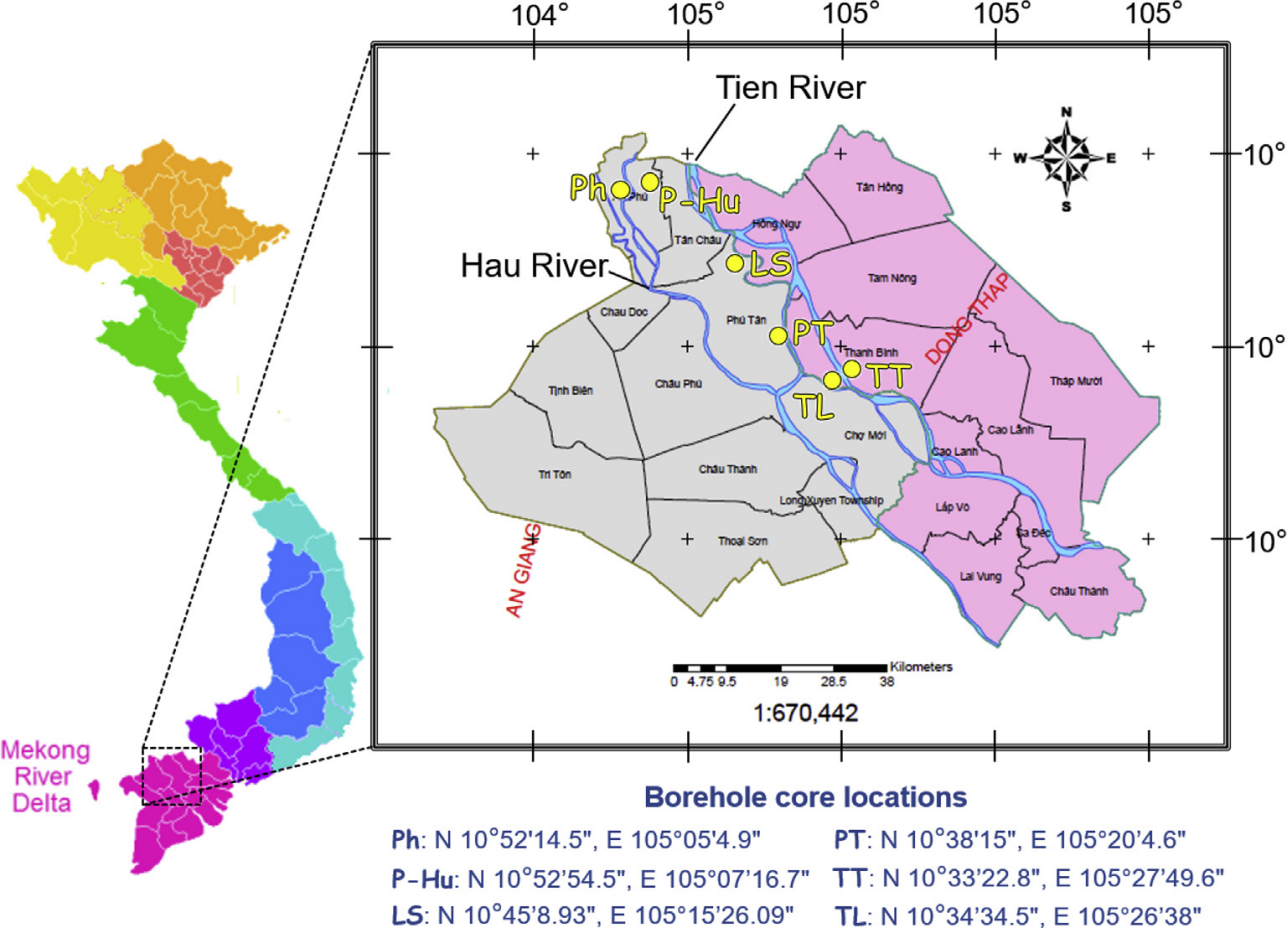
A map of Vietnam superimposed with the locations of borehole cores used in this study [1].

**Fig. 2:**
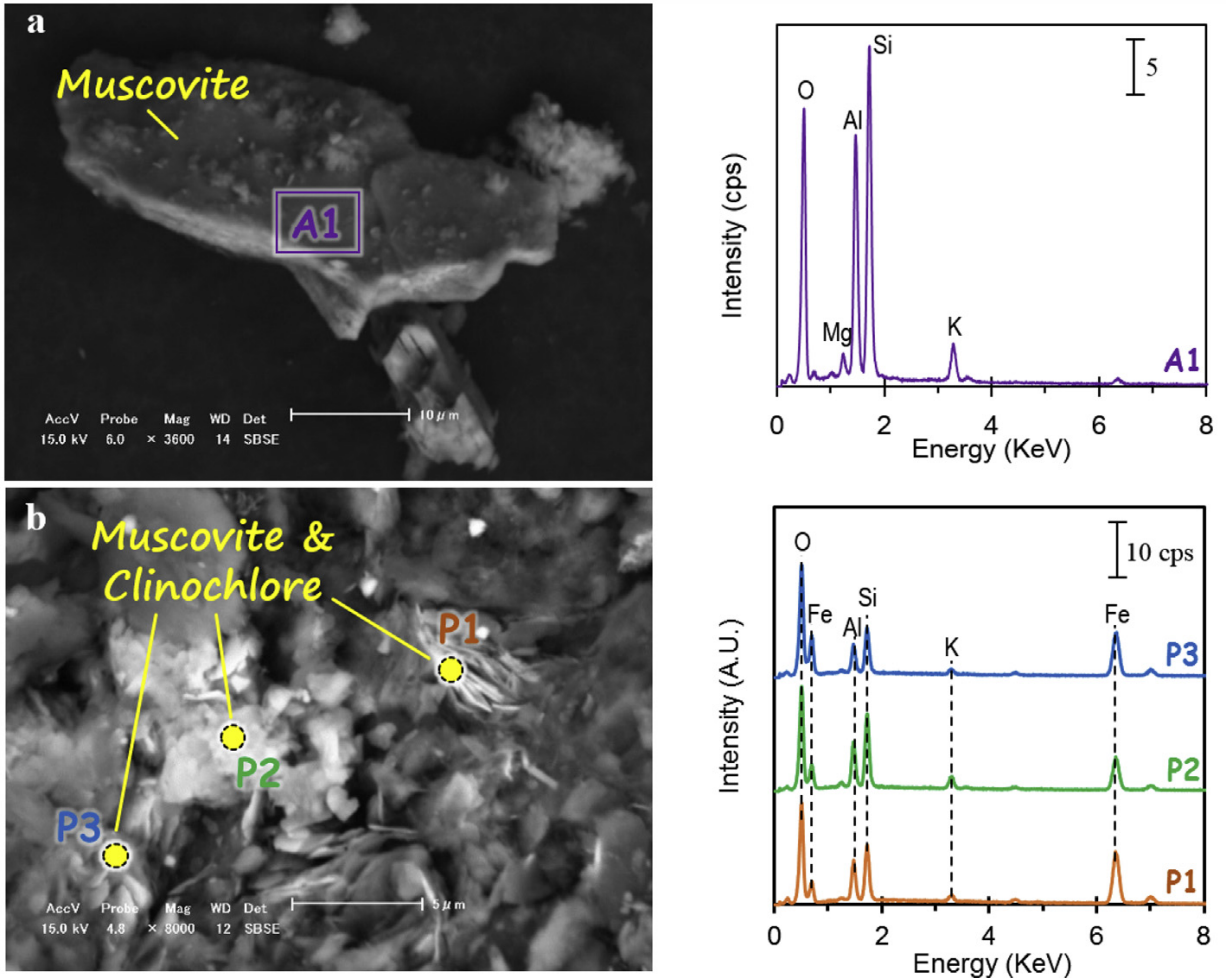
(a) SEM photomicrograph and EDX area spectrum of muscovite, and (b) SEM photomicrograph and EDX point spectra of muscovite and clinochlore.

**Fig. 3:**
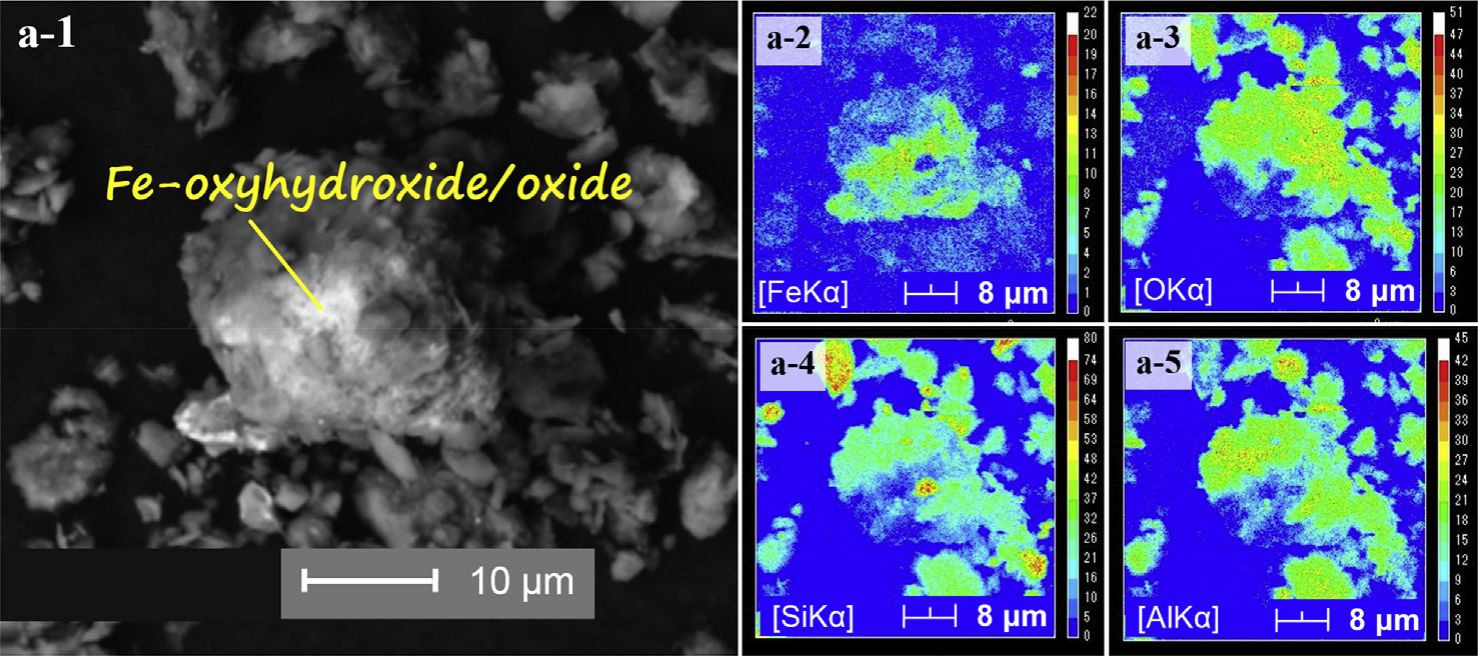
SEM photomicrographs of Fe-oxyhydroxide/oxide (a-1) and the corresponding elemental maps of Fe (a-2), O (a-3), Si (a-4), and Al (a-5).

**Fig. 4:**
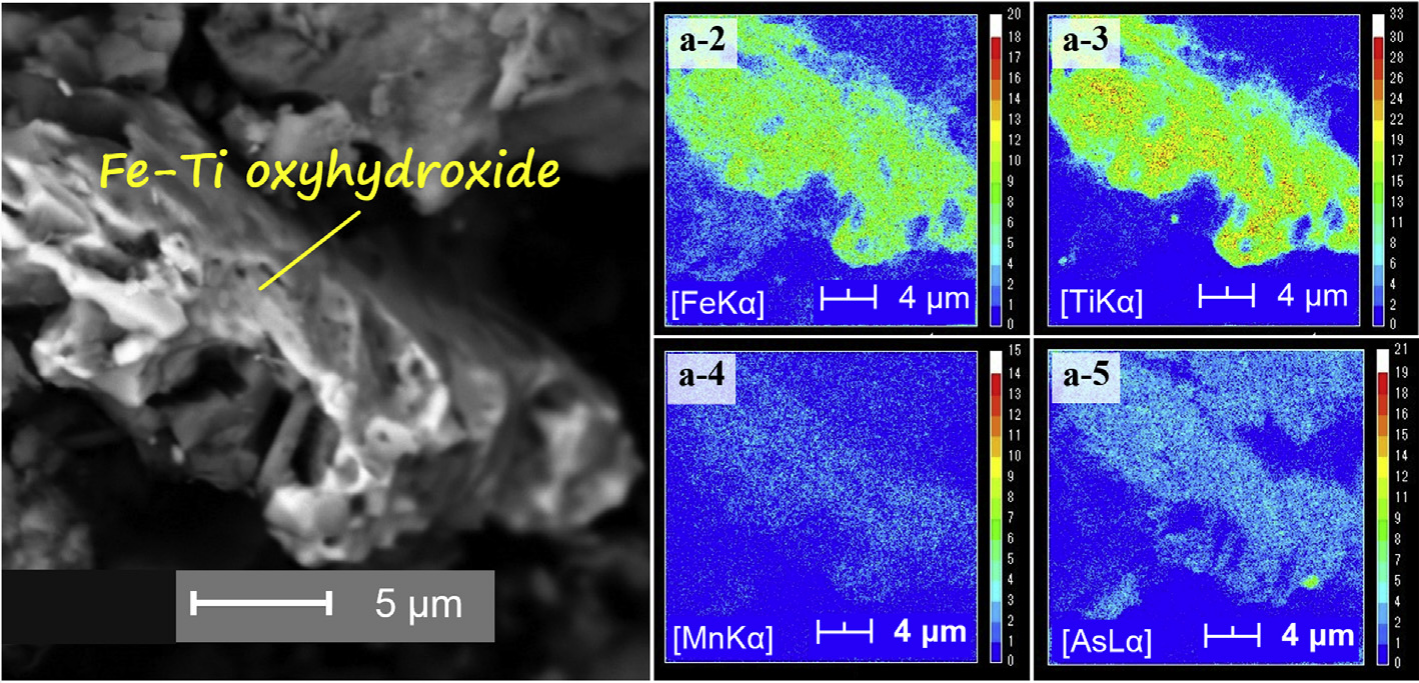
SEM photomicrographs of a Fe-oxyhydroxide/oxide particle-containing Ti, Mn and As (a-1) and the corresponding elemental maps of Fe (a-2), Ti (a-3), Mn (a-4), and As (a-5).

**Fig. 5:**
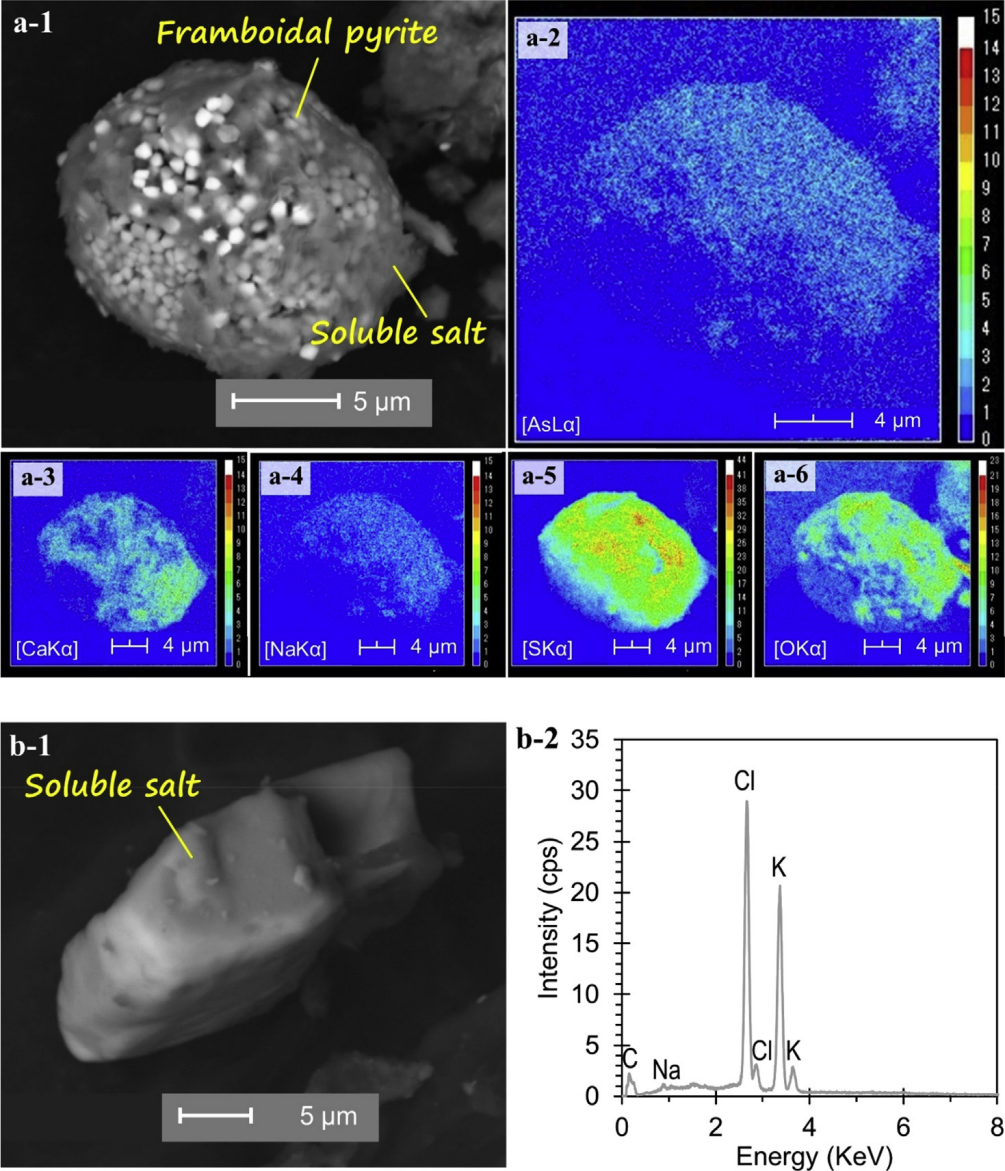
SEM photomicrographs of As-bearing soluble salt and framboidal pyrite (a-1) and the corresponding elemental maps of As (a-2), Ca (a-3), Na (a-4), S (a-5), and O (a-6), and SEM photomicrographs of a salt-like particle composed mainly of K and Cl (b-1), and the EDS spectrum of the particle (b-2).

**Fig. 6:**
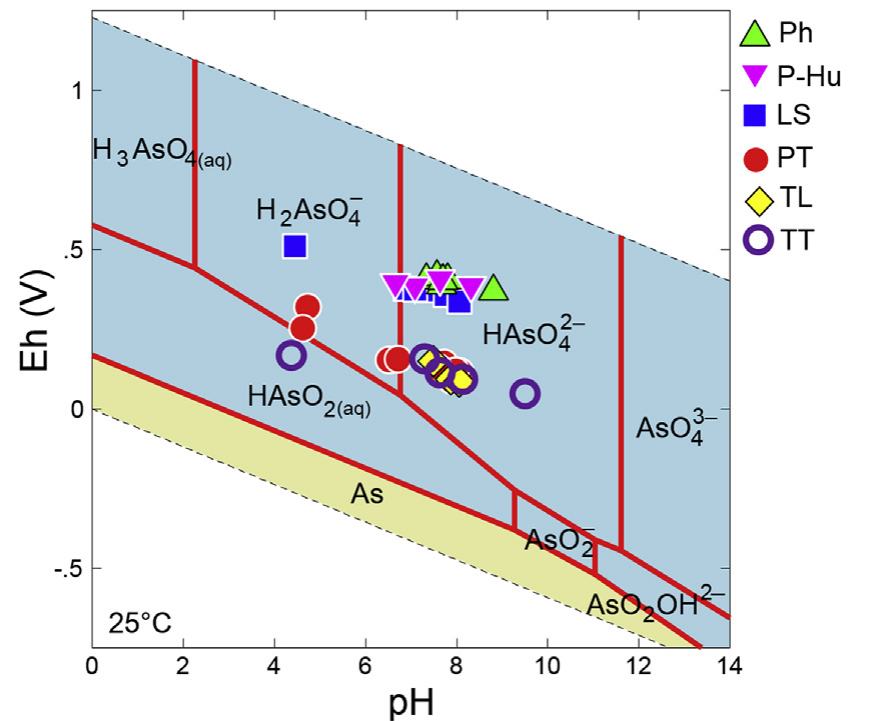
The Eh-pH predominance diagram of As at 25 °C, 1.013 bars, and activity of As = 10^−4^. Data points represent the actual leachate pH and Eh values measured during the leaching experiments.

The dataset describes the chemical and mineralogical properties of distinct geological features found in six borehole cores collected up to a depth of 40 m in the Vietnamese-side of the Mekong Delta (Fig. 1; Table 1, Table 2, Table 3, Table 4, Table 5). It also includes scanning electron photomicrographs and energy dispersive spectra and/or elemental maps of phyllosilicates like muscovite and Clinochlore, including iron/aluminum oxyhydroxides, framboidal pyrite and soluble salts containing arsenic (As) as illustrated in Fig. 2, Fig. 3, Fig. 4, Fig. 5. Thirty-four sediment samples from the six borehole cores were leached and the geochemical properties of the leachates were determined including the concentrations of major cations and anions, dissolved heavy metals and organic matter, arsenic, and other important components like aluminum (Al), dissolved Si and nitrate (NO_3_^−^) (Table 6). Finally, arsenic speciation is described using an Eh-pH diagram created from the measured solute activities of As in the leachates (Fig. 6).

**Table 1:** The lithology of sediment samples from the six borehole cores.

Sample name	Lithology
LS-1	Grey clayey silt
LS-2	Dark grey fine sand with thin brownish grey clayey silt
LS-3	Alternating dark grey fine sand and brownish grey clayey silt (ratio 1:1)
LS-4	Brownish grey clayey silt
LS-5	Grey fine sand (distributed sample)
LS-6	Brownish grey clayey silt
Ph-1	Brownish grey clayey silt
Ph-2	Brownish grey clayey silt, partly including sand
Ph-3	Brownish grey fine sand with thin clayey silt layers
Ph-4	Brownish grey clayey silt
Ph-5	Brownish grey clayey silt, some parts with grey fine sand layers
Ph-6	Brownish grey clayey silt
P-Hu-1	Brownish grey clayey silt and sandy silt
P-Hu-2	Brownish grey clayey silt and sandy silt with a thin grey fine sand layer
P-Hu-3	Grey fine sand (distributed sample)
P-Hu-4	Grey fine sand
PT-1	Grey brownish to reddish clayey silt
PT-2	Dark grey clay with some reddish clayey silt layers
PT-3	Dark grey clay with thin sand layers with some organic matter
PT-4	Dark grey clay with thin sand layers
PT-5	Dark grey clay with fine sand layers
PT-6	Black clayey peat
PT-7	Dark grey fine sand
PT-8	Black clayey peat
PT-9	Reddish-grey clayey containing laterite
PT-10	Dark grey coarse sand
TL-1	Grey clayey silt
TL-2	Dark grey fine sand
TL-3	Dark grey coarse sand
TT-1	Grey-yellowish clayey silt with dark-grey clayey silt layers
TT-2	Dark-grey clayey silt
TT-3	Grey fine sand
TT-4	Grey medium sand
TT-5	Grey coarse sand

**Table 2:** The chemical compositions of sediment samples from the six borehole cores.

Sample	SiO_2_	Al_2_O_3_	Fe_2_O_3_	MnO	MgO	CaO	Na_2_O	K_2_O	P_2_O_5_	S	As	TOC	LOI
	wt.%	wt.%	wt.%	wt.%	wt.%	wt.%	wt.%	wt.%	wt.%	mg/kg	mg/kg	wt.%	wt.%
LS-1	51.4	15.2	4.64	0.02	1.06	0.23	0.16	2.45	0.02	14	17.7	1.13	6.21
LS-2	58.0	9.18	4.94	0.11	1.13	0.54	<0.13	2.13	0.02	4.56	10.3	1.07	4.05
LS-3	53.8	10.3	4.86	0.09	1.29	0.60	0.20	2.14	0.03	3.18	9.20	0.85	4.18
LS-4	46.6	14.0	7.01	0.14	1.69	0.57	<0.12	2.52	0.05	4.41	19.1	1.40	7.10
LS-5	68.2	6.02	2.52	0.03	0.71	0.35	0.30	1.88	0.01	1.49	4.10	0.17	1.41
LS-6	47.0	14.7	6.95	0.13	1.75	0.47	0.18	2.68	0.06	2.21	15.5	1.11	6.79
Ph-1	42.6	13.1	9.55	0.08	1.19	1.26	<0.12	2.28	0.08	0.49	16.6	0.28	5.30
Ph-2	50.9	11.9	5.36	0.10	1.12	0.63	0.17	2.11	0.05	0.71	12.7	0.97	4.91
Ph-3	57.8	9.45	4.13	0.05	0.96	0.41	0.29	1.99	0.03	1.54	7.20	0.49	2.73
Ph-4	55.0	10.9	4.70	0.09	1.06	0.52	<0.12	2.09	0.04	1.87	12.4	0.78	4.18
Ph-5	49.6	13.3	5.85	0.11	1.26	0.54	<0.12	2.29	0.05	0.82	15.8	1.08	5.75
Ph-6	48.2	13.0	6.03	0.11	1.26	0.59	0.37	2.23	0.05	0.94	13.0	1.04	5.82
P-Hu-1	49.1	13.2	6.43	0.12	1.49	0.91	0.30	2.49	0.06	0.55	15.4	1.03	5.72
P-Hu-2	48.2	13.7	6.74	0.15	1.35	0.58	<0.12	2.56	0.06	0.77	15.9	1.05	5.71
P-Hu-3	71.2	4.41	2.35	0.03	0.37	0.29	<0.13	1.68	0.00	0.44	4.40	0.04	0.90
P-Hu-4	70.7	3.60	2.13	0.02	0.29	0.23	0.40	1.64	0.01	0.44	5.70	0.02	0.77
PT-1	70.8	23.2	4.42	0.06	2.10	0.30	0.31	1.70	0.12	340	7.65	0.36	4.24
PT-2	66.2	24.3	7.45	0.01	1.48	0.18	0.41	2.59	0.03	1251	16.9	0.40	6.44
PT-3	72.4	17.1	4.94	0.08	2.10	0.55	0.78	2.26	0.06	12240	11.7	0.96	4.75
PT-4	66.2	22.1	7.17	0.15	2.69	0.72	0.61	2.88	0.13	2316	14.0	0.89	6.31
PT-5	86.3	9.61	2.34	0.03	1.33	0.43	1.05	1.82	0.08	819	4.38	0.16	1.01
PT-6	62.9	22.8	7.74	0.05	3.54	0.74	1.33	2.68	0.15	39420	32.5	12.8	28.6
PT-7	84.0	11.6	2.00	0.03	1.95	0.46	1.58	1.62	0.11	607	2.78	0.22	26.5
PT-8	64.9	25.4	4.48	0.02	2.58	0.42	0.86	3.24	0.10	5415	33.0	6.61	16.2
PT-9	67.7	21.6	7.43	0.12	2.17	0.45	0.77	2.77	0.11	169	13.1	0.18	5.13
PT-10	90.0	7.28	2.03	0.02	0.92	0.33	0.96	1.58	0.06	189	2.70	0.07	1.14
TL-1	71.5	19.1	5.84	0.13	2.30	0.88	0.73	2.55	0.17	312	15.8	0.77	4.67
TL-2	81.6	12.5	3.49	0.05	1.71	0.54	1.24	2.20	0.09	65.9	7.39	0.08	1.85
TL-3	91.3	6.87	1.73	0.03	0.72	0.25	0.83	1.37	0.07	9.38	4.97	0.04	0.84
TT-1	68.4	21.8	4.77	0.03	2.22	0.77	0.80	2.18	0.08	67.7	12.1	0.07	5.29
TT-2	75.8	17.8	3.79	0.03	1.84	0.44	0.76	2.39	0.08	835	16.0	0.68	3.66
TT-3	89.4	8.27	1.83	0.02	0.99	0.26	0.98	1.30	0.04	52.8	3.49	0.05	1.14
TT-4	90.2	7.68	1.74	0.02	0.83	0.22	1.00	1.41	0.06	39.1	2.32	0.05	1.07
TT-5	84.2	9.50	4.55	0.11	0.89	0.26	0.92	1.93	0.11	147	10.4	0.14	1.72

**Table 3:** The relative abundances of minerals in sediments from borehole cores Ph and P-Hu.

Sample	Depth (m)	Relative abundance (wt.%)											
		Qz	An	Ab	Kln	Ms	Clc	Cal	Dol	Py	Gt	Hem	Gp
Ph-1	2.8–3.0	60.9	13.1	0.6	6.8	4.8	10.2	2.8	–	0.3	0.5	–	–
Ph-2	7.2–7.4	64.7	14.4	–	2.4	9.7	6.9	–	1.5	–	0.1	0.3	–
Ph-3	11.6–11.8	79	13.1	–	2.4	1.7	1.7	0.4	0.5	–	1.6	–	–
Ph-4	14.7–14.9	71.9	17.9	–	0.4	6	2.7	–	0.3	–	0.2	0.7	–
Ph-5	16.2–16.4	68.1	8.3	1.6	4.8	12.2	4.3	–	0.2	0.1	–	0.3	–
Ph-6	19.1–19.3	68.4	10.3	1.1	5	11.3	3	–	–	–	0.7	0.3	–
P-Hu-1	3.2–3.4	58.4	11.7	0.2	5.2	13.6	7	–	1.9	0.2	0.7	1	–
P-Hu-2	6.2–6.4	59	10.7	1.5	6.4	16	4.2	–	1.4	–	–	0.8	–
P-Hu-3	10.7–10.9	73.5	10.3	2.4	0.4	3	9.2	–	1.1	–	–	0.1	–
P-Hu-4	16.7–16.9	86.4	10.4	–	1	2.2	–	–	–	–	–	–	–

Note: Qz: Quartz; An: Anorthite; Ab: Albite; Ms: Muscovite; Kln: Kaolinite; Clc: Clinochlore; Cal: Calcite; Dol: Dolomite; Py: Pyrite; Gt: Goethite; Hem: Hematite; Gp: Gypsum; "–": Not detected.

**Table 4:** The relative abundances of minerals in sediment samples from borehole cores LS and PT.

Sample	Depth (m)	Relative abundance (wt.%)											
		Qz	An	Ab	Kln	Ms	Clc	Cal	Dol	Py	Gt	Hem	Gp
LS-1	3.2–3.4	64.1	8.3	–	5.4	14.1	3.7	–	–	2.2	1.9	–	0.3
LS-2	5.8–6.0	73.9	8.2	7.6	–	6.6	2.6	–	–	–	0.3	0.4	0.4
LS-3	7.7–7.9	72.2	18.9	–	3.1	1.1	3.8	0.3	–	0.6	–	–	–
LS-4	10.2–10.7	59.8	8	–	8.5	14.5	5.7	–	1.4	–	–	1.7	0.4
LS-5	13.7–13.9	72.9	18.6	1.2	3.3	3.8	–	–	–	0.2	–	–	
LS-6	18.7–18.9	56.9	5.7	1.9	7.2	18	5.7	–	2.4	–	1.7	–	0.5
PT-1	1.2–1.3	61.6	7.5	2.8	6.1	13.8	5.5	–	–	–	2.7	–	–
PT-2	4.2–4.3	57.2	5.7	2.9	10.3	7.4	12.4	1.1	–	–	1.7	–	1.1
PT-3	6.4–6.5	71.6	15.3	–	4.8	0.7	4.8	0.7	–	1	1.1	–	–
PT-4	11.2–11.3	57.2	13.8	–	10.1	0.8	9	1.6	3.4	1.4	2	0.7	–
PT-5	17.6–17.7	81.2	17.4	–	0.3	1.1	–	–	–	–	–	–	–
PT-6	19.2–19.3	39.8	10.1	6.6	11.7	6	15.2	0.8	–	6	–	1.2	4.3
PT-7	21.6–21.7	80.1	16.1	1.2	1.2	–	0.1	–	–	–	–	1.4	–
PT-8	24.5–24.6	41.2	2.7	2.7	14.2	22.1	11	0.5	2.4	2.3	–	–	0.9
PT-9	28.3–28.4	59.5	14.8	–	12.3	3.8	5.6	–	2.7	1.3	–	–	–
PT-10	38.1–38.2	82.4	15.4	–	1	1.2	–	–	–	–	–	–	

Note: Qz: Quartz; An: Anorthite; Ab: Albite; Ms: Muscovite; Kln: Kaolinite; Clc: Clinochlore; Cal: Calcite; Dol: Dolomite; Py: Pyrite; Gt: Goethite; Hem: Hematite; Gp: Gypsum; "–": Not detected.

**Table 5:** The relative abundances of minerals in sediment samples from borehole cores TL and TT.

Sample	Depth (m)	Relative abundance (wt.%)											
		Qz	An	Ab	Kln	Ms	Clc	Cal	Dol	Py	Gt	Hem	Gp
TL-1	3.4–3.5	60.3	16.3	0.1	6.6	2.9	12.6	0.6	–	0.6	–	–	–
TL-2	9.5–9.6	64.8	17.8	–	5	3.1	4.8	0.7	3.3	0.6	–	–	–
TL-3	16.5–16.6	88.9	4	6.2	–	–	–	–	–	–	–	0.8	–
TT-1	2.4–2.5	61.1	6.2	–	7.7	19.5	2.9	0.8	–	–	0.1	1.6	–
TT-2	3.4–3.5	76.5	13.7	–	4.5	0.8	4.1	0.1	–	0.1	–	0.2	–
TT-3	12.4–12.5	87.4	9.4	1.9	0.5	0.8	–	–	–	–	–	–	–
TT-4	25.3–25.4	85.2	12.3	1	–	1.1	–	0.4	–	–	–	–	–
TT-5	37.7–37.8	85.2	12.2	–	–	2.6	–	–	–	–	–	–	–

Note: Qz: Quartz; An: Anorthite; Ab: Albite; Ms: Muscovite; Kln: Kaolinite; Clc: Clinochlore; Cal: Calcite; Dol: Dolomite; Py: Pyrite; Gt: Goethite; Hem: Hematite; Gp: Gypsum; "–": Not detected.

**Table 6:** Geochemical properties and chemical compositions of leachates from sediments of the six borehole cores.

Sample	pH	EC	Eh	Temp.	Na	K	Mg	Ca	Fe	Al	Si	As	Cl	NO_3_^−^	SO_4_^2-^	HCO_3_^−^	DOC
		mS/m	mV	°C	mg/l	mg/l	mg/l	mg/l	mg/l	mg/l	mg/l	μg/l	mg/l	mg/l	mg/l	mg/l	mg/l
LS-1	4.45	37.5	511	19.2	3.78	3.74	11.8	48.1	1.14	1.25	1.68	3.26	2.72	0.41	179	–	4.86
LS-2	6.88	23.3	377	19.1	3.92	4.07	12.9	21.0	0.52	1.84	3.88	0.82	2.72	0.17	91.9	14.6	2.70
LS-3	7.41	21.3	372	16.0	5.00	8.22	11.8	14.5	3.29	9.84	14.7	0.67	11.4	0.34	62.7	17.7	3.67
LS-4	7.76	21.1	357	19.7	12.9	8.75	15.2	6.24	11.1	33.5	50.0	3.0	6.74	0.48	66.6	20.7	9.78
LS-5	7.09	11.9	373	15.3	1.67	2.32	7.43	8.97	1.55	4.54	7.48	0.13	1.74	–	43.6	6.71	3.49
LS-6	8.06	28.2	336	19.6	41.4	18.3	12.1	2.26	38.8	10.9	158	6.36	20.9	11.7	63.7	28.7	20.2
Ph-1	8.80	12.7	371	10.3	3.41	0.72	5.06	16.7	0.19	0.36	2.40	0.62	1.63	3.05	3.20	72.6	4.49
Ph-2	7.66	6.83	390	10.2	2.54	3.28	1.62	3.01	7.58	24.0	28.4	4.50	4.14	0.21	3.58	22.6	15.0
Ph-3	7.33	4.77	406	9.80	1.64	2.58	2.57	4.58	7.91	18.9	23.6	2.22	4.34	0.32	7.88	8.54	6.16
Ph-4	7.56	5.06	416	11.1	4.54	2.62	1.91	3.43	6.32	17.0	21.1	3.24	3.39	0.36	5.07	14.6	10.8
Ph-5	7.68	6.08	406	11.4	10.0	3.75	2.18	1.88	11.9	28.9	39.8	5.92	1.96	0.33	7.28	14.0	17.1
Ph-6	7.68	9.84	404	10.3	10.1	3.50	3.83	6.18	9.19	23.1	32.4	4.08	12.7	0.26	10.5	19.5	10.7
P-Hu-1	8.30	11.6	389	11.5	5.84	3.16	2.62	12.4	3.85	10.6	13.4	3.35	3.90	0.73	5.01	60.4	10.5
P-Hu-2	7.62	7.37	410	11.3	5.01	2.42	1.54	3.20	5.04	11.8	15.7	2.58	7.79	1.90	5.69	13.4	11.7
P-Hu-3	7.07	2.16	387	15.3	1.26	0.89	0.41	1.88	0.88	0.66	1.76	1.08	2.21	–	1.69	9.76	2.63
P-Hu-4	6.64	1.39	398	15.5	0.98	0.60	0.19	1.00	0.38	0.28	1.40	0.11	1.57	–	1.11	5.49	2.98
PT1-1	7.72	0.24	146	26.0	30.6	10.9	8.03	8.09	34.8	66.5	92.1	9.27	30.1	–	50.5	18.1	6.04
PT1-2	4.72	0.30	322	25.2	17.0	2.18	11.7	16.6	0.45	1.10	4.34	1.08	14.5	–	112	1.46	1.42
PT1-3	6.50	0.55	155	24.6	15.5	4.26	31.7	42.6	–	0.16	2.56	0.66	6.32	–	228	9.76	2.99
PT1-4	8.07	0.23	119	24.6	22.0	14.1	13.5	7.85	26.0	74.4	118	4.52	13.1	10.3	42.9	39.1	13.0
PT1-5	7.60	0.13	130	24.9	7.48	6.07	4.65	6.72	5.71	12.0	18.8	2.19	8.03	–	33.5	11.2	3.96
PT1-6	4.61	2.17	255	24.4	128	22.4	149	125	1.14	0.19	6.88	44.2	51.0	–	1134	N.D.	15.4
PT1-7	7.97	0.13	122	24.4	8.75	5.56	3.93	6.47	5.84	12.2	18.3	3.18	9.87	–	27.4	15.6	5.20
PT1-8	6.60	0.56	158	24.7	84.0	42.5	21.3	6.62	65.1	301	424	49.1	62.3	17.1	119	13.2	56.1
PT1-9	7.89	0.15	91	24.9	27.9	42.6	22.2	5.46	121	343	491	14.7	18.0	22.1	4.72	36.1	11.8
PT1-10	7.59	0.08	132	24.6	5.24	2.89	2.14	4.04	1.75	1.85	3.99	0.98	7.74	–	15.5	9.76	4.80
TL2-1	7.86	0.08	95	25.0	4.61	2.78	2.22	8.87	5.37	11.4	17.5	6.14	7.70	–	4.78	34.2	6.04
TL2-2	8.05	0.03	88	25.0	0.93	1.52	0.74	3.35	3.23	2.63	4.70	2.64	1.05	1.22	2.46	17.1	2.24
TL2-3	7.48	0.03	151	25.0	1.26	1.66	0.85	2.84	3.31	2.38	4.59	4.43	2.54	–	2.06	14.2	1.72
TT3-1	9.49	0.24	50	24.5	49.5	40.4	23.2	9.37	138	289	411	45.9	3.20	1.06	9.59	132	6.06
TT3-2	7.62	0.09	116	24.5	18.3	12.8	6.69	4.94	33.4	100	145	31.2	9.19	–	17.3	17.1	44.1
TT3-3	7.28	0.14	159	24.9	19.3	2.45	1.01	1.21	3.33	3.23	5.76	2.36	36.1	–	4.52	8.30	43.4
TT3-4	4.36	0.16	170	24.8	18.6	2.04	0.59	0.94	0.66	0.80	2.81	0.81	39.4	–	3.51	N.D.	88.5
TT3-5	8.12	0.14	95	24.9	19.2	5.42	2.92	1.08	42.8	40.1	63.4	20.0	31.0	–	3.18	15.6	4.41

Note: “–” means not detected.

## 2 Experimental design, materials, and methods

### 2.1 Study area, sampling locations, and a brief description of sediment samples

The study area is located in the An Giang and Dong Thap Provinces of Vietnam, which are notorious for their As-contaminated groundwater. Fig. 1 is a map of Vietnam showing where the six borehole cores with depths of 20–40 m were collected, and Table 1 provides lithological descriptions of the sediments with depth. A total of 34 samples were obtained from each distinct lithological feature of the borehole cores. The particle size distribution of sediments varied with depth and ranged from clayey silt to coarse sand. Two peat layers were also observed in one of the borehole cores (PT). Groundwater levels (GWL) in the borehole core sampling locations were shallow (0.3–3.6 m below the surface), and the groundwater samples were predominantly under oxidizing conditions (Eh values measured on-site were between +0.18 and +0.45 V *vs* SHE).

### 2.2 Chemical and mineralogical compositions of distinct lithological features in the borehole cores

Thirty-four sediment samples were air-dried in the laboratory, sealed in polypropylene (PP) bottles and then shipped to Hokkaido University, Japan for analyses. The chemical and mineralogical analyses were carried out on pressed powders of samples ground to <50 μm with an agate mortar. The chemical analysis was determined by X-ray fluorescence spectroscopy (XRF) (SpectroXepos, Rigaku Corporation, Japan) while the mineral components were identified by X-ray powder diffraction (XRD) (MultiFlex, Rigaku Corporation, Japan). All 34 XRD patterns were analyzed using Match!^®^ (Crystal Impact, Germany) to identify minerals and semi-quantitatively determine their relative abundances in the samples. Loss on ignition (LOI) was determined by gravimetry, which was done by heating a known amount of sample in a muffle furnace at 750 °C for 1 h after drying at 110 °C in an oven for 24 h. The loss in mass of the sample corresponded to LOI. The total organic carbon (TOC) of the sediments was measured using a solid sample combustion unit attached to a total carbon analyzer (TOC-V_CSH_-SSM-5000A, Shimadzu Corporation, Japan). Selected samples that have exceptionally high As were also examined using a scanning electron microscope with energy dispersive X-ray spectroscopic capability (SEM-EDX) (Superscan SSX-550, Shimadzu Corporation, Japan). The chemical compositions of the sediments are listed in Table 2 while the identified minerals and their approximate relative abundances in the sediments are summarized in Table 3, Table 4, Table 5

Photomicrographs of minerals and phases important in the geochemical evolution of groundwater and As mobility like clays [1], phyllosilicates (Fig. 2), organic matter [1], iron oxyhydroxides (Fig. 3), iron-titanium oxyhydroxides (Fig. 4) as well as pyrite and soluble salts (Fig. 5) are also shown [2], [3], [4], [5], [6], [7], [8], [9], [10], [11].

### 2.3 Geochemical properties and chemical compositions of leachates from the sediments

The leaching experiment was based on the standard Japanese leaching test for contaminated soils (Environmental Agency of Japan Notification No. 46) [12] and was done by mixing 15 g of sediment samples (<2 mm) with 150 ml of deionized (DI) water at 200 rpm using a reciprocal shaker for 6 h. The temperature, pH, Eh and electrical conductivity (EC) of suspensions were measured after 6 h and the liquid (supernatant) and solid (residue) were separated by centrifugation for 30 minutes at 3500 rpm. The supernatant was decanted and filtered through 0.45 μm Millex^®^ membrane filters (Merck Millipore, USA) prior to the chemical analyses. The concentrations of dissolved iron (Fe), manganese (Mn), silicon (Si) and aluminum (Al) were determined by inductively coupled plasma atomic emission spectroscopy (ICP-AES, ICPE-9000, Shimadzu Corporation, Japan) while the concentrations of major coexisting cations (Ca^2+^, Mg^2+^, Na^+^ and K^+^) and anions (Cl^−^, NO_3_^−^, SO_4_^2−^) were quantified by ion chromatography (ICS-90 and ICS-1000, Dionex Corporation, USA). Dissolved As concentrations greater than 0.1 mg/l were analyzed directly by ICP-AES while leachates with less than 0.1 mg/l of As were first pretreated and then analyzed using a hydride-vapor generation unit connected to an ICP-AES (detection limit: 0.1 μg/l; uncertainty = ±5%). For the pretreatment of leachates with As concentrations less than 0.1 mg/l, 10 ml of leachate was mixed with 5 ml of 12 M HCl, 0.67 ml of 20% potassium iodide (KI), 0.67 ml of DI water, and 0.33 ml of 10% ascorbic acid solution, and this mixture was allowed to equilibrate for 3 h prior to the chemical analysis [13]. Dissolved organic carbon (DOC) was measured by a total carbon analyzer (TOC-V_CSH_, Shimadzu Corporation, Japan) while HCO_3_^−^ concentrations were estimated from the alkalinity and pH using PHREEQC [14]. The alkalinity was measured by titration of a known volume of leachate with 0.02 N sulfuric acid (H_2_SO_4_) solution until pH 4.8 [15], [16]. The standard ICP-AES and ion chromatography have margins of error of around 2%.

The geochemical properties of the leachates, including the concentrations of As, Si, Al, dissolved heavy metals, DOC, and coexisting ions, are summarized in Table 6. The pH ranged from 4 to 10 and the concentrations of coexisting ions varied with depth and sampling location. The speciation of As in the leachates is plotted in an Eh-pH diagram created by the Geochemist's Workbench^®^ [17] based on the measured solute activities (Fig. 7). Except for one sample from TT, As in the leachates exist as various oxyanions of arsenate (As^V^).
